# Effects of Farming System on the Rheological Behavior of Rennet-Induced Coagulation in Milk from Skopelos Breed Goats

**DOI:** 10.3390/foods14081316

**Published:** 2025-04-10

**Authors:** Kali Kotsiou, Marios Andreadis, Georgios Manessis, Athina Lazaridou, Costas G. Biliaderis, Zoitsa Basdagianni, Ioannis Bossis, Thomas Moschakis

**Affiliations:** 1 Department of Food Science and Technology, School of Agriculture, Aristotle University of Thessaloniki, 54124 Thessaloniki, Greece; kkotsiou@agro.auth.gr (K.K.); mandr@agro.auth.gr (M.A.); athlazar@agro.auth.gr (A.L.); biliader@agro.auth.gr (C.G.B.); 2 Department of Animal Production, School of Agriculture, Aristotle University of Thessaloniki, 54124 Thessaloniki, Greece; gmane@agro.auth.gr (G.M.); basdagianni@agro.auth.gr (Z.B.); bossisi@agro.auth.gr (I.B.)

**Keywords:** goat farming system, Skopelos breed, coagulation properties, dynamic rheometry, goat milk composition

## Abstract

This study examined the impact of extensive and intensive farming systems on the rheological behavior of rennet-induced goat milk coagulation in the indigenous Greek Skopelos goat breed. Milk samples were obtained from Skopelos dairy goats reared under extensive and intensive farming systems at two farms in Greece. Rennet-induced coagulation kinetics (at 35 °C) and curd rheological properties were assessed using dynamic rheometry. Milk from the extensive system exhibited longer rennet coagulation time (RCT) but resulted in curds with higher storage modulus (G′) and complex viscosity (η*), indicating formation of stronger coagulated structures compared to curds from the intensive system. The differences can be attributed to variations in milk composition and the structural characteristics of protein components, e.g., casein fractions, between the two systems. Principal component analysis (PCA) revealed that the farming systems could be differentiated based on the rheological properties of the curds, specifically on structure-related parameters (loss tangent, tanδ, apparent yield stress, τ_y_). Strong correlations (*p* < 0.01) were noted between G′max and caseins/total proteins (positive), as well as with pH (negative), in both farming systems. These findings offer valuable insights into animal farming practices and cheese production, providing evidence of the relationships between goat-rearing systems and rheological properties of rennet-coagulated milk products.

## 1. Introduction

Lately, consumers’ preference for goat milk products has surged due to increasing awareness of their health benefits and concerns over cow’s milk association with allergies and other gastrointestinal ailments [1]. Compared to cow milk, goat milk contains lower levels of α_s1_-casein, a protein strongly associated with allergic reactions to cow milk consumption [2]. Additionally, the biologically active components of goat milk, such as peptides, oligosaccharides, short- and medium-chain fatty acids, and phospholipids, play a key role in preventing chronic diseases—particularly cardiovascular diseases—primarily due to their antioxidant properties while also supporting gastrointestinal health [3,4]. Different goat-farming systems might influence milk composition, including protein and fat content. Moreover, differences in bioactive compounds, which are related to oxidative stability and distinctive sensory characteristics like polyphenols, volatile compounds, and specific fatty acids have been reported when comparing milk from different farming systems [5,6,7,8]. The variations and occasional discrepancies in reported data highlight the combined influence of farming systems (extensive, semi-intensive, and intensive) and management practices, including feeding, breeding, housing, and animal health, on milk composition [7]. For instance, grazing-based goat-farming systems produced milk with higher total protein and casein content, attributed to the diverse and high-quality forage compared to hay-based diets [9]. Additionally, natural pasture-based systems have been shown to produce goat milk richer in fat, health-beneficial nutrients (e.g., fatty acids, vitamins), volatile compounds (e.g., flavor, terpenes), and in some cases, higher protein content [8,10]. In contrast, intensive farming systems relying on concentrate-based diets, yielded milk richer in protein but lower fat content [7]. Furthermore, intensive systems often achieve a more consistent milk composition compared to forage-based systems due to controlled feeding regimens [11].

These compositional differences not only affect raw milk quality but also have a strong impact on its rheological behavior during cheese production and, consequently, on consumer preferences. In fact, the ability of milk to form stable curds during coagulation is fundamental in cheesemaking, since the rheological properties of these gel network structures are important determinants of texture, yield, and overall quality of the cheese. In goat milk, the predominance of β-casein over α_s1_-casein, combined with lower protein and fat content, reduced κ-casein, and larger casein micelles, results in weaker protein interactions. Thus, goat milk curds tend to be softer, more fragile, and yield less cheese which also exhibits compromised textural integrity compared to cow milk, suggesting that modifying milk composition could improve curd quality [12]. Indeed, higher casein content resulted in improved curd firmness, while increasing protein content delayed the rennet coagulation time (RCT) in goat milk [13]. Beyond compositional features, pH and somatic cell counts (SCCs), which are related to udder health, also influence milk coagulation kinetics due to their strong positive correlations with RCT [14]. It is evident that farming and/or feeding systems can influence milk composition, which in turn affects curd formation. As a result, curd rheological properties could be linked to different farming systems. In a recent study, goats from intensive farming systems produced milk with higher concentrations in protein and fat, while milk from extensive farms showed faster RCT but slower curd firming, lower curd firmness, and reduced fat and energy recoveries [10]. However, the authors highlighted that the farming systems could be differently influenced by the type of goat breed. Since the animal genetic makeup affects milk nutrient content and specific breeds are typically associated with certain farming systems, much of the existing literature focuses on the breed effects on rheological properties, rather than the direct influence of farming systems [10,15]. Genetic polymorphisms in goat milk proteins, particularly in casein genes, have been reported to influence protein expression levels, thereby affecting milk rheology and cheese yield, with αs1-casein playing a crucial role in determining these functional properties [16].

Greece holds the leading position in dairy goat farming within the European Union (EU-27), with a reported 2,747,700 goats, a staggering 26.63% of the total goat population in the EU, according to FAO [17]. In Greece, goat milk is primarily utilized for the production of traditional cheeses by taking advantage of its chemical composition as a critical factor in the cheesemaking industry. As such, the quality of goat milk plays a pivotal role in determining the sensory characteristics, yield, and overall quality attributes of the cheese, thereby becoming an important determinant for the success of the dairy industry. Dairy goat farming in Greece is characterized by a diverse range of genetic populations [18] which are raised in a variety of farming systems, ranging from intensive to extensive. The Skopelos breed, one of the most important goat breeds in Greece, is renowned for its exceptional adaptability to dry and hot climates, making it well suited to the country’s challenging environmental conditions. The annual milk yield of the Skopelos breed ranges from 250 to 400 kg per 210-day lactation period, with prolificacy ranging from 1.2 to 1.6 kids per doe.

The present study aimed to evaluate the impact of extensive and intensive farming systems on the rheological properties of goat milk curds from the indigenous Greek Skopelos breed. The study focuses on milk coagulation behavior and curd strength and explores the relationships between farming system type, milk composition and cheesemaking performance. To achieve this, the kinetics of rennet-induced coagulation and the mechanical properties of the resulting curds were assessed using dynamic rheometry. The findings of the current work could provide valuable insights into optimizing cheese production processes and improving curd quality and yield, thus offering benefits for both industrial and artisanal cheese producers. This study is the first detailed investigation into the cheesemaking performance of goat milk from the Skopelos breed raised under different farming systems, addressing the growing demand for indigenous goat milk products and their potential for producing high-quality cheese. A key strength of this study is the elimination of breed as a confounding factor largely influencing milk composition and cheesemaking performance. Both goat populations, raised under the extensive and intensive farming systems, originated from the same genetic pool on a farm on Skopelos island, ensuring that the observed differences are directly attributable to farming practices rather than genetic variation.

## 2. Materials and Methods

### 2.1. Milk Samples Collection and Preparation

Milk samples were collected from healthy, Greek-indigenous purebred Skopelos dairy goats reared under either extensive or intensive farming systems. All animals were in their second or third lactation and had similar parturition dates. The annual milk yield for this breed ranges from 250 to 450 kg over a 210-day lactation, with prolificacy between 1.2 and 1.6 kids per doe. Sampling was performed during morning milking—yielding 0.85 ± 0.01 kg and 0.98 ± 0.01 kg for the extensive and intensive systems, respectively—once per lactation, after weaning, over two consecutive lactation periods (February 2022 and February 2023). The two farms were located in Greece: the intensive farm (AGRO) was located in the Attika region, and the extensive farm (SKOPELOS) on Skopelos Island. On the AGRO farm, goats were permanently housed in well-ventilated stables with controlled temperature and humidity, and straw bedding. Animals were fed 1.0–1.2 kg/day of commercial concentrate and 0.9–1.8 kg/day of alfalfa hay, under a high-input management system with no access to grazing. On the other hand, the SKOPELOS farm followed a low-input pastoral management system, characterized by year-round grazing on natural grasslands, shrublands, and woodlands, with limited infrastructure and labor. Goats were housed indoors at night in permanent shelters with ventilation openings, absorbent flooring, and sufficient space per animal. They were hand-milked and received 0.5–1.0 kg/day of supplementary concentrate and 0.0–0.5 kg/day of alfalfa hay during winter months when pasture availability was reduced.

Milk samples were collected in 250 mL screw-capped plastic flasks and promptly cooled to 4–6 °C using ice packs. The samples were transported in insulated containers to the laboratory, where analyses were performed within 24 h. Before coagulation testing, the milk was pasteurized at 63 °C for 30 min, and pH measurements were conducted immediately after pasteurization. A total of 162 milk samples (Intensive 2022, *n* = 39; Intensive 2023, *n* = 45; Extensive 2022, *n* = 29; Extensive 2023, *n* = 49) were tested for their coagulation behavior following rennet addition.

### 2.2. Milk Compositional Analysis

Chemical composition (fat, total solids, total proteins, and casein content) was determined with a Foss MilkoScan FT-plus fully automatic milk FTIR analyzer (Foss Electric A/S, Hillerød, Denmark) in accordance with the ISO/IDF method [19]. Somatic cell counts (SCCs) were determined using a Fossomatic 5000 (Foss Electric A/S, Hillerød, Denmark) instrument.

### 2.3. Rheological Measurements of Rennet-Induced Milk Coagulation

**Figure 1:**
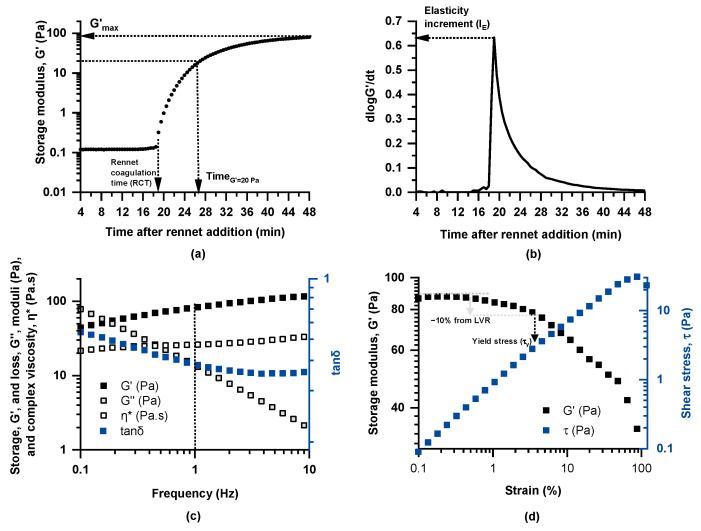
Representative curves obtained through dynamic rheological measurements and graphical representation of the calculated parameters: (**a**) coagulation kinetics (strain 0.5%, frequency 1 Hz) following rennet addition (1 g/100L milk) at 35 °C and (**b**) curve of log G′(t) slope versus time upon coagulation of Skopelos breed goat milk, as well as (**c**) frequency sweep (strain 0.5% at 35 °C) and (**d**) strain sweep (frequency 1 Hz at 35 °C) tests of the formed curds.

Dynamic (oscillatory) rheological testing of milk coagulation kinetics at 35 °C following rennet addition was performed in a Physica MCR 300 rheometer (Physica Messtechnik GmbH, Stuttgart, Germany) with a concentric cylinder geometry (CC27, diameter of cup and bob, 28.92 and 26.66 mm, respectively). Specifically, rennet (CHY-MAX^®^ Powder Extra NB, Chr. Hansen A/S, Hørsholm, Denmark) and calcium chloride were added to 50 mL of milk (using amounts corresponding to 1 g and 15 g per 100 L of milk, respectively), preheated to 35 °C in a water bath under gentle stirring. The timer was set to zero at the moment of rennet addition, and stirring continued for 1 min to ensure complete dispersion. Subsequently, the milk sample was transferred to the rheometer, and measurements began 3 min after rennet addition to ensure consistency across tests. To avoid water loss during measurements, a thin layer of paraffin oil was applied over the samples. Coagulation kinetics were determined by applying small deformation oscillatory testing at a constant strain of 0.5% and a frequency of 1 Hz for 45 min at 35 °C, while the storage (G′) and loss (G′′) moduli and loss tangent (tan δ = G′′/G′) were continuously monitored (Figure 1a); the experimental set-up and protocols adopted in rheological testing were essentially as described by Lazaridou et al. [20]. For characterization of the coagulation properties, the following rheological parameters were calculated: rennet coagulation time (RCT) (min), defined as the time at which G′ exhibits an abrupt increase; elasticity increment (I_E_ = (dlogG′/dt)_max_) as an index of coagulation rate (Figure 1b); time to reach G′ = 20 Pa (T_G′=20Pa_); and maximum storage modulus (G′_max_) (Pa) determined as the value of G′ at the end of the coagulation kinetics measurement (Figure 1a).

For evaluation of the viscoelastic properties of the formed curds, following the coagulation process (48 min after rennet addition), small deformation oscillatory measurements were performed over the frequency range of 0.1–10 Hz (mechanical spectra), at a constant strain of 0.5% and temperature of 35 °C, while G′, G′′, tanδ and complex viscosity (η*) were monitored (Figure 1c). Finally, strain sweeps were performed at a constant frequency of 1 Hz and 35 °C in the range of 0.1–100% of applied deformation (Figure 1d). Finally, the apparent yield stress, *τ*_y_, of the samples was determined as the stress (Pa) at which the storage modulus (G′) values start to show a deviation of 10% from the linear viscoelastic region, LVR.

### 2.4. Statistical Analysis

Differences between the farming systems were identified using a *t*-test. Levene’s test, stepwise linear regression analysis (SLRA), and Pearson correlations were performed using the IBM SPSS Statistics software (Version 29). Principal component analysis (PCA) was performed and visualized in RStudio (Build 394), using the FactoMineR (Version 2.11), factoextra (Version 1.0.7), ggplot2 (Version 3.5.1) and corrplot (Version 0.95) packages.

## 3. Results and Discussion

### 3.1. Milk Compositional Characteristics

**Figure 2:**
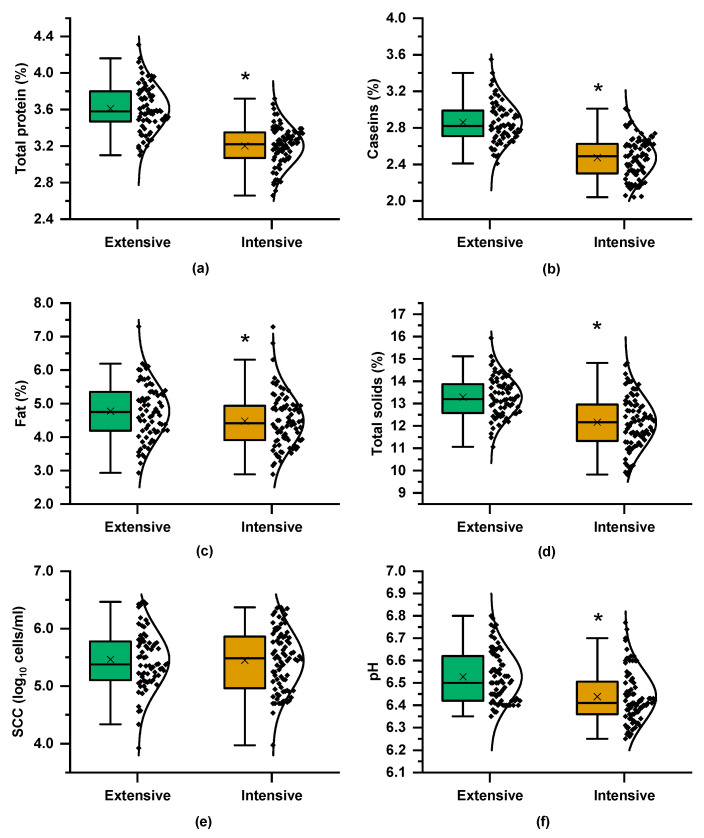
Boxplots and distribution of compositional and quality parameters of the milk samples from the Skopelos breed goats raised under extensive and intensive farming systems: (**a**) total protein content, (**b**) caseins, (**c**) fat, (**d**) total solids, (**e**) somatic cells count (SCC), and (**f**) pH; *t*-test; * = *p* < 0.05.

As shown in Figure 2, milk from the extensive farming system had higher total protein, fat, and, consequently, total solids compared to the intensive system. The higher total protein content also resulted in significantly greater casein levels. Regarding udder health traits, milk from the extensive system exhibited higher pH values, while SCCs remained similar between both systems. The compositional differences can be attributed to the influence of farming practices, particularly the feeding strategy. The extensive system, which relied on natural grazing, most likely provided a more diverse and nutrient-rich forage, contributing to increased protein and casein content. In contrast, goats in the intensive system were fed on typical commercial concentrates, alfalfa hay, and wheat straw, resulting in comparatively lower levels of these components. This concurs with previous findings that linked grazing-based diets to enhanced milk composition due to the superior nutritional quality of pasture-based feed [7,9].

### 3.2. Rennet Coagulation Kinetics

Dynamic rheological measurements were conducted to study the kinetics of milk coagulation at 35 °C following rennet addition. The obtained curves were characterized by a sharp increase in the G′ values with time followed by a “plateau” region (Figure 1a and Figure S1). Initially, *t*-tests were performed within each farming system to determine whether the milk-sampling year influenced the measured coagulation parameters RCT, T_G′=20Pa_, I_E_, and G′_max_ (Tables S1 and S2). The results showed no significant differences between the two sampling years for either the intensive or the extensive farming system. Furthermore, Levene’s test confirmed that the variance in these parameters remained consistent throughout the years.

**Figure 3:**
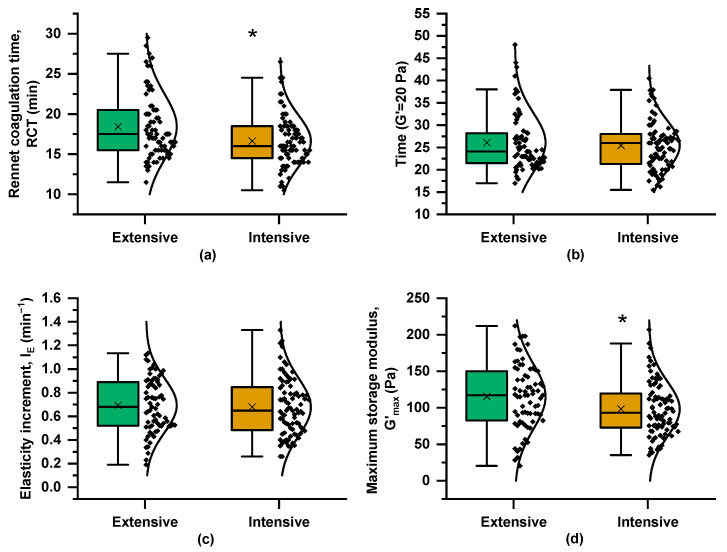
Boxplots and distribution of rheological parameters derived from curves of rennet-induced coagulation kinetics (as in Figure 1) of milk samples from the Skopelos breed goats raised under extensive and intensive farming systems: (**a**) rennet coagulation time (RCT), (**b**) time when G′ was equal to 20 Pa (T_G′=20Pa_), (**c**) elasticity increment (I_E_), and (**d**) maximum storage modulus (G′_max_); *t*-test; * = *p* < 0.05.

Figure 2 presents boxplots of the coagulation parameters and their variances across the two farming systems. RCT was significantly shorter in milk from the intensive system, although samples from both systems reached G′ = 20 Pa at nearly the same time and they exhibited similar I_E_ values (Figure 3b,c). However, G′_max_, derived from the “plateau” phase of the coagulation curve, differed significantly between the two systems, with mean values of 115 Pa for the extensive system curds and 98 Pa for milk samples from the intensive farming scheme. The coagulation properties are influenced not only by milk composition, including protein and fat content, but also by pH level and SCC [14]. The greater variability in milk coagulation properties from the extensive system (Figure 3) may partly reflect the broader variation in compositional and other quality traits (Figure 2).

In a recent study, intensive systems yielded milk higher in protein and fat, while extensive systems showed faster RCT but slower curd firming and lower curd firmness, whereas the two animal-rearing systems were largely influenced by different goat breeds [10]. In another work comparing pasture (cultivated or rangeland) and hay-based (high- and low-quality hay, goats kept indoors) feeding systems of Norwegian dairy goats, the coagulation properties were not significantly influenced by the feeding treatment but were negatively affected when milk was collected during the late grazing season [9]. In the present study, the variation in the coagulation performance in the extensive system was further amplified by the inclusion of non-coagulating milk samples. In fact, five out of seventy-eight samples either did not coagulate or failed to reach a G′ value of 20 Pa by the end of the measurement (Figure S1). However, this behavior could not be related to the tested compositional and quality characteristics of the individual non-coagulating milks. Similarly, in a recent study on milk from the Slovenian Alpine goat breed 4.1–23.6% of samples were reported as exhibiting slow coagulation and 0.8–10.8% as showing non-coagulating behavior across three samplings conducted over a four-year period. In the latter case, the slow and non-coagulating milk samples exhibited higher fat and protein content along with significantly higher SCCs compared to normal coagulating samples [21]. Beyond compositional and milk quality-related characteristics, genetic factors and especially casein polymorphism are also associated with coagulation properties and might explain the observed variability in the rheological responses [22,23].

Pearson’s coefficients of correlation were calculated for evaluation of possible relationships among the different variables (Table 1). It was indicated that in both farming systems, coagulation time-related parameters (RCT and T_G′=20Pa_) were primarily associated with the initial pH of the milk, with higher pH values corresponding to longer RCT and an extended curd formation period; the latter kinetic parameter was also positively correlated to the SCC in the case of the intensive farming system. Additionally, caseins followed by total protein content were influential factors, showing significant positive correlations with RCT in the extensive system and negative correlations with T_G′=20Pa_ in the intensive system. Strong correlations (*p* < 0.01) were also noted between G′_max_ values and caseins and total proteins (positive), as well as pH (negative) in both systems. Finally, the SCC and TS showed weaker correlations (*p* < 0.05) with G′_max_, being negatively and positively correlated, respectively, but only in the intensive system, while in both systems, I_E_ was only negatively correlated with the pH values.

Furthermore, SLRA was applied to examine the statistical significance of each independent variable of the coagulation process in a linear model (Table 2). Regarding RCT, it was shown that other than pH, which contributed the most to the total variance of the target variable in both systems, fat and protein content improved the linear regression models for the intensive and extensive system, respectively. For the prediction of the T_G′=20Pa_ values, TS was assigned as the second predictor alongside the pH in the intensive system, whereas SLRA analysis did not identify any main contributors other than pH for the I_e_ values in either system. In addition, SLRA showed that pH followed by the total protein content were the two variables contributing to G′_max_ prediction in the extensive system, accounting for 32.8% of the total variability. On the other hand, G′_max_ prediction in the intensive system reached up to 53.9% when caseins, pH, and TS were included in the model. It should be noted that G′_max_, an indicator of gel strength, was not only influenced by the quality characteristics of the milk but also showed a negative correlation with RCT (Table 1); however, only compositional and quality characteristics were included in the SLRA analysis. This implies that shorter coagulation times resulted in firmer curds at the end of the measurement. Similar behavior has been recorded when using cow and sheep milk for cheesemaking, and it was attributed to the fact that these measurements capture two successive stages of the coagulation process [24,25].

The importance for milk coagulation kinetics of compositional features, such as fat, protein and casein contents, and other quality characteristics, like pH and SCC, related to udder health status is well established. Depending on the goat breed, high protein and casein content delays the coagulation process but on the other hand results in an increased curd-firming rate and curd firmness [26,27]. Additionally, since caseins are the primary contributors to coagulation and account for most of the milk protein content, their correlation with total protein is very high (Table 1), and when each is separately included in statistical models, the contribution patterns to coagulation parameters are nearly identical, providing a further indication of their interconnected roles [27]. Regarding fat content, its positive effect on RCT is well established, as milk flocculation involves the collision and aggregation of fat droplets, which facilitate the formation of the protein network during rennet coagulation [28]. In the present study, fat showed insignificant correlations with rennet coagulation kinetics parameters (Table 1); however, it appeared to enhance the SLRA prediction models for the RCT variable in the intensive system (Table 2), suggesting a complementary effect. In fact, Stocco et al. [27] concluded that when fat content affects cheesemaking, it modulates the correlated effects of protein and casein contents. Regarding health-related traits, a lower milk pH is known to reduce RCT and to accelerate curd-firmness development by dissolving the micellar colloidal calcium phosphate, decreasing the electrostatic repulsion between casein micelles, as well as facilitating dissociation of caseins from micelles and thereby enhancing rennet activity [29,30,31]. Similarly, in the present study, pH was indicated as the most critical determinant of coagulation kinetics within the examined range of compositional characteristics since it was strongly correlated with all the tested parameters, confirming the results reported in literature for various types of milk [32,33,34]. On the other hand, a high SCC has been found to prolong rennet clotting time [35]. In the present study, weak correlations between SCC and certain rennet coagulation kinetics parameters were observed only in the intensive system samples (Table 1); however, the SCC did not contribute to the prediction of these parameters in the SLRA (Table 2). Overall, while the coagulation process was primarily regulated by pH, differences in the contributions of other traits between the two systems suggested that, to a lesser extent, coagulation may be influenced by the farming system, likely due to variations in feeding practices. Indeed, the indirect effect of diet on RCT through casein content and type has been previously reported [9].

### 3.3. Viscoelastic Properties of Goat Milk Curds

**Figure 4:**
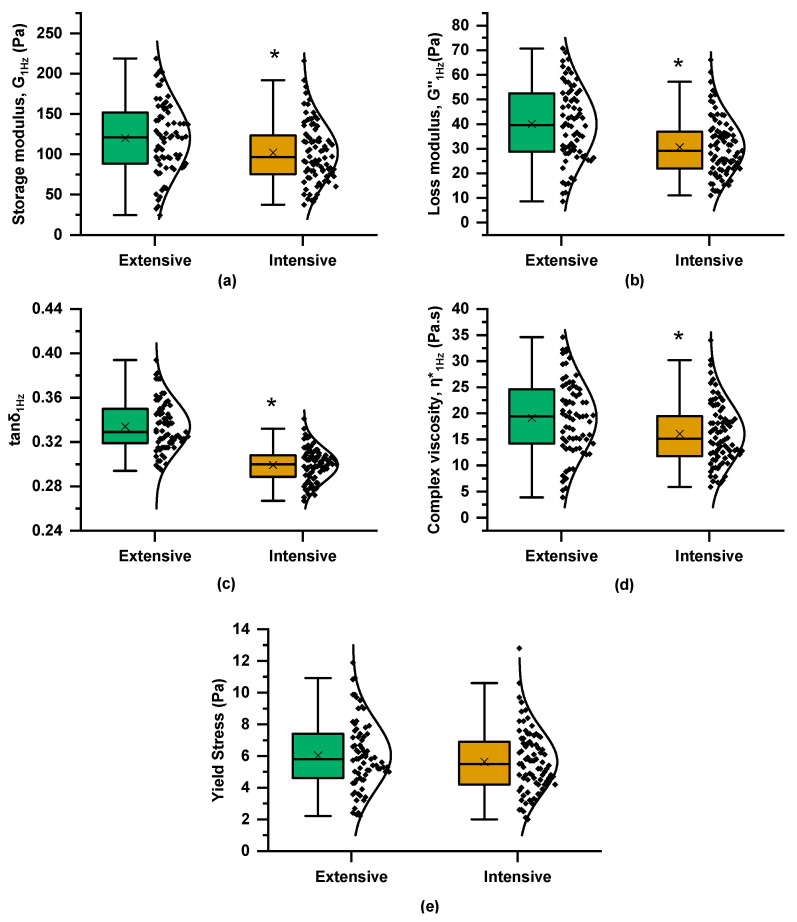
Boxplots and distribution of rheological characteristics of rennet-induced curds (48 min after rennet addition) from the Skopelos breed goats of extensive and intensive farming systems derived from the frequency (strain 0.5% at 35 °C) and strain (frequency 1 Hz at 35 °C) sweep tests (as in Figure 1): (**a**) storage modulus at 1 Hz, G′_1Hz_; (**b**) loss modulus at 1 Hz, G″_1Hz_; (**c**) loss tangent at 1 Hz; tanδ_1Hz_; (**d**) complex viscosity at 1 Hz, η*_1Hz_; and (**e**) apparent yield stress (τ_y_); *t*-test; * = *p* < 0.05.

The mechanical spectra of the curds, obtained 48 min after rennet addition, revealed that the G′ values were higher than G″ across the entire frequency range, displaying the characteristics of viscoelastic materials with a relatively weak coagulated structure, which was evidenced by the moderate frequency dependence of both dynamic moduli and the tanδ values (0.1 < tanδ < 1) (Figure 1c). The mean and median values of G′_1Hz_, G″_1Hz_, and η*_1Hz_ were higher in the curds produced by milks from the extensive farming system (Figure 4), pointing to their ability to form firmer curds compared to those obtained from the intensive system. Instead, the lower average tanδ_1Hz_ values in the intensive system indicate a stronger elastic response. The higher total protein and fat content in milk from the extensive system, compared to the intensive system, resulted in an overall increase in TS (Figure 2), which may become entrapped within the casein network (composite structure), enhancing the curd’s hardness at the expense of its elasticity. Studies examining the effect of farming systems on the rheological properties of curds are limited, with breeding and feeding factors being closely interlinked and strongly influencing the outcomes. For instance, a study conducted in Sardinia, Italy, reported that milk from extensive farms, predominantly sourced from a local goat breed, exhibited lower curd firmness compared to milk from intensive farms, which were dominated by a cosmopolitan specialized dairy breed [10]. Another study suggested that outdoor pasture and indoor hay feeding had a weaker impact on curd firmness compared to the influence of the grazing season [9].

G′_1Hz_, G″_1Hz_, η*_1Hz_, and tanδ were highly correlated (*p* < 0.01) with total protein, caseins, pH, TS, and SCC, with Pearson correlations varying between the examined variables depending on the farming system (Table 1). For instance, G′_1Hz_, G″_1Hz_, and η*_1Hz_ showed positive correlations (*p* < 0.01) with total protein and caseins for curds from both farming systems, being stronger (higher r) for the intensive system compared to the respective r values of the extensive. On the other hand, pH was negatively related to these rheological parameters exhibiting the opposite trend (r values for the extensive system were higher than those of the intensive). The above parameters were correlated (*p* < 0.05) with TS and SCC only in the intensive system with the exception of G″_1Hz_ that was correlated with TS for both farming schemes. Finally, the tanδ showed stronger (positive) correlations with total protein and casein in the extensive system compared to the intensive animal-rearing system. Regarding the contribution of each independent variable to the outcome of G′_1Hz_, G″_1Hz_, and η*_1Hz_, SLRA (Table 2) revealed that these rheological parameters could be better predicted in the intensive system, with caseins, pH, and TS contributing 54.6–55.2% of the total variation for the outcome. In contrast, in the extensive system, total protein and pH explained approximately 34.0–37.3% of the predicted G′_1Hz_, G″_1Hz_, and η*_1Hz_ values. The results also indicated that the tanδ values of the curds from the intensive system were mostly influenced by the casein content followed by SCC, while for the extensive system, total protein content was the main predictor, with pH being as the secondary factor. However, given the strong intercorrelation between total protein and caseins, it can be inferred that casein, followed by pH, are the actual contributors in the extensive system, with the model prioritizing total protein due to statistical redundancy.

Overall, the viscoelastic properties of the curds seemed to be better predicted for the group of milks of the intensive system, which could be attributed to the lower variability in compositional and quality characteristics of these samples. On the other hand, the higher dynamic moduli and complex viscosity values of the extensive system curds (Figure 4a,b,d), possibly related to the higher total protein content and TS (Figure 2a,d), imply stronger casein networks and can potentially result in better cheese yield, greater retention of solids in the composite gel matrix, and improved texture; such responses in the physical properties of the milk curds are essential for maintaining the structural integrity of the coagulated system during cheesemaking [36,37,38]. All these parameters linked with the curd firmness are important determinants of quality and yield of cheese and thus of the overall economic returns [26].

### 3.4. Apparent Yield Stress of Goat Milk Curds

A rennet-induced milk coagulum remains stable when undisturbed but undergoes rapid syneresis if it is cut or broken. In fact, syneresis is an essential step in cheesemaking, induced by cutting of the curd into smaller pieces, to accelerate the syneresis rate and foster moisture removal. An enhanced tendency for rennet coagulum to undergo syneresis is linked to network rearrangements following gelation (van Vliet et al., 1991 [39]). A lower yield stress of cheese curd indicates a softer, less stable structure compared to curds with higher yield stress. Systems with low yield stress typically indicate weaker gel network structures and are often associated with higher moisture content of the curds, negatively impacting curd firmness and final cheese quality. The mean *τ*_y_ values, estimated by the strain sweep test of the curds (Figure 1d), did not reveal any differences between the two farming systems, regardless of their differences in the viscoelastic properties of the curd (Figure 4e). Pearson correlations showed that this parameter was highly correlated with coagulum firmness, as reflected by the G′, G′′, and η* values from the frequency sweep test, showing also a positive correlation with caseins and total proteins and a negative correlation with the initial pH of the milk (Table 1). SLRA indicated that caseins accounted for 47.5% of the *τ*_y_ values’ variance in the intensive system, with the model improving to 53.3% when pH was also included (Table 2). On the other hand, for the extensive system, only the total protein content was included in the model, explaining 26.5% of the yield stress variability. Given the known intercorrelation between total protein and casein contents, it is reasonable to assume that casein is a key factor in the rheological responses of the curds in both systems. These findings highlight the distinct drivers of τ_y_ in the two systems, with caseins playing a clearer role in the intensive system, while the extensive system’s prediction remained lower probably due to inherent variability in the samples of this system. The role of proteins, especially in the form of casein, has been well known to affect curd strength and therefore syneresis, with rearrangements of particles in the coagulum network structure being largely responsible for this phenomenon [38]. Regarding pH, lower values decrease electrostatic repulsion and promote aggregation, leading to a stable network that entraps fat and other solids [32,40].

### 3.5. Principal Component Analysis

**Figure 5:**
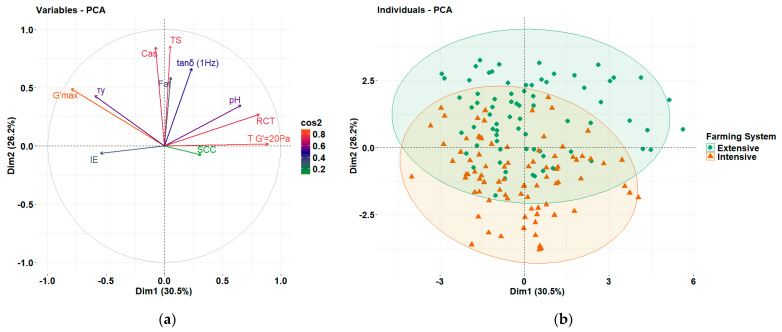
(**a**) Factor loadings and (**b**) sample distribution in the 2-dimensional coordinate system, derived from principal component analysis (PCA) applied to compositional, quality, and rheological variables of milk and curd samples from the Skopelos breed goats raised in Greece under extensive and intensive farming systems.

Pearson correlation analysis and SLAR identified some links between the rennet coagulation parameters and the farming system, which are primarily driven by the compositional characteristics as discussed above. For a better understanding, PCA was conducted using the compositional and quality characteristics of the milk, as well as the rheological properties derived from rennet coagulation kinetics and the evaluation of the resulting curds to evaluate the cheesemaking characteristics of milks from the two different systems. To ensure unique contributions to the component structure/matrix (and to avoid over-representing highly correlated metrics), the most representative variables were selected among the highly interrelated data sets. For example, RCT was selected over T_G′=20Pa_, as these variables were highly correlated; i.e., earlier coagulation onset corresponded to the curd reaching a G′ value of 20 Pa sooner (Table 1). From the frequency sweep test parameters, G′ was excluded as it is equivalent to G′_max_, while G′′ and η* were excluded due to their high correlation with G′ (and G′_max_). Among the compositional characteristics, casein was selected over total proteins content due to its direct involvement in gel network formation during cheesemaking. PCA extracted four components with eigenvalue above 1 (Table 3), with the first and second components explaining 56.7% of the variance. RCT, G′_max_, T_G′=20Pa_, I_E_, and *τ*_y_ along with pH were strongly associated with PC1 (Table 3 and Figure 5a), highlighting the critical role of pH in rennet-mediated coagulation kinetics and its strong relationship with the process time-related parameters. On the other hand, the compositional characteristics, such as fat, casein, and TS, were loaded onto PC2 and positively corelated with G′_max_, tanδ_1Hz_, and *τ*_y_, justifying their importance on curd structure and emphasizing their importance in curd structure by influencing gel strength, interactions and rearrangement potential among the interconnected particles in the composite gel protein network structures, and syneresis. Finally, PC3 consisted only of compositional features, while I_E_ and tanδ_1Hz_ were also loaded to PC4 along with SCC (Table 3).

As illustrated by the score plot of the first two principal components (Figure 5b), the extensive system predominantly clustered above the x-axis (PC2), while the samples of the intensive system clustered below, indicating a potential differentiation between the two farming systems based on the tanδ_1Hz_ and τ_y_ values, related to the elastic response of the curds and their structural stability, both being influenced by the compositional characteristics. More specifically, curds from the extensive system milk shifted towards stronger structures, which could potentially lead to an increased cheese yield. The overlap noted between samples of the two groups in the middle suggests that certain samples exhibit common structural or network behaviors. The similar spread of both systems along PC1 indicated that rheological parameters related to the coagulation kinetics could not differentiate the two systems as they were primarily affected by pH. Overall, the PCA revealed that although milk coagulation kinetics did not fully distinguish the samples between the two systems due to their strong dependence on pH, their compositional differences led to distinct structural characteristics in the resultant curds, which are crucial for cheese yield and moisture retention by the curds.

## 4. Conclusions

This study investigated the influence of two farming systems (extensive and intensive) of the Skopelos goat breed on rennet-induced coagulation kinetics and curd rheological properties. Dynamic rheological measurements showed that pH played a dominant role in coagulation parameters such as RCT and G′_max_ in both systems. Moreover, higher total protein and casein contents were associated with enhanced curd strength, particularly in milk from the extensive system. This indicates that milk from extensive systems forms curds with stronger casein networks, which could potentially translate into improved cheese yield and texture, despite exhibiting greater variability. SLRA models indicated that casein and pH were critical predictors of the curd properties, while SCC played a secondary role. Principal component analysis (PCA) suggested that, although coagulation kinetics were primarily influenced by pH, differences in the elastic properties of the curds (as reflected by tanδ and yield stress, *τ*_y_) offer a means to differentiate between the two farming systems. However, some overlap noted between the two sample clusters implies common traits between the systems. These findings not only advance our understanding of how farming practices affect milk coagulation properties but also provide practical insights for the dairy industry. By linking specific compositional and rheological traits to cheesemaking performance, the study underscores the importance of integrating research findings into practice. Such evidence-based approaches can guide producers in optimizing feeding strategies and processing conditions to optimize cheese yield, texture, and end-product quality. Overall, this work illustrates how farming practices can improve practical cheesemaking processes, ultimately contributing to better product outcomes in both artisanal and industrial environments.

## Figures and Tables

**Table 1:** Pearson correlation coefficients (r) between the tested traits of milks from the Skopelos breed goats raised in Greece under extensive (lower triangle) and intensive (upper triangle) farming systems.

	Fat	TP ^1^	Cas	TS	SCC	pH	RCT	T_G′=20Pa_	G′_max_	IE	G′_1Hz_	G″_1Hz_	tanδ_1Hz_	η*_1Hz_	τ_y_
Fat		−0.199	0.061	0.920 **	−0.047	0.339 **	−0.090	−0.058	0.085	−0.154	0.081	0.076	0.018	0.081	0.009
TP	0.095		0.856 **	−0.247 *	−0.144	−0.219 *	0.028	−0.247 *	0.589 **	0.076	0.602 **	0.615 **	0.261 *	0.603 **	0.599 **
Cas	0.140	0.859 **		−0.222 *	−0.176	−0.141	0.042	−0.222 *	0.648 **	0.061	0.661 **	0.676 **	0.324 **	0.662 **	0.689 **
TS	0.839**	0.466 **	0.461 **		−0.088	0.379 **	−0.065	−0.100	0.234 *	−0.153	0.231 *	0.231 *	0.103	0.231 *	0.147
SCC	0.026	−0.037	−0.115	0.022		0.180	0.183	0.264 *	−0.277 *	−0.154	−0.275 *	−0.233 *	0.199	−0.271 *	−0.289 **
pH	0.026	−0.190	−0.056	−0.102	0.174		0.502 **	0.501 **	−0.377 **	−0.324 **	−0.370 **	−0.356 **	0.035	−0.369 **	−0.336 **
RCT	−0.026	0.283 *	0.349 **	0.115	0.028	0.480 **		0.863 **	−0.490 **	−0.255 *	−0.465 **	−0.431 **	0.208	−0.462 **	−0.267*
T_G′=20Pa_	−0.026	0.099	0.167	0.018	0.045	0.448 **	0.922 **		−0.686 **	−0.335 **	−0.667 **	−0.635 **	0.113	−0.664 **	−0.412 **
G′_max_	−0.052	0.426 **	0.352 **	0.198	−0.199	−0.456 **	−0.575 **	−0.699 **		0.356 **	0.999 **	0.989 **	0.178	0.999 **	0.770 **
IE	0.052	−0.110	−0.087	−0.118	−0.164	−0.335 **	−0.498 **	−0.566 **	0.429 **		0.352 **	0.340 **	0.001	0.352 **	0.091
G′_1Hz_	−0.139	0.451 **	0.376 **	0.204	−0.209	−0.452 **	−0.547 **	−0.679 **	0.999 **	0.415 **		0.990 **	0.187	1.000 **	0.777 **
G″_1Hz_	0.049	0.525 **	0.435 **	0.248 *	−0.191	−0.406 **	−0.493 **	−0.639 **	0.980 **	0.403 **	0.982 **		0.315 **	0.992 **	0.735 **
tanδ_1Hz_	0.055	0.457 **	0.357 **	0.251 *	0.067	0.247 *	0.354 **	0.269 *	−0.047	−0.048	−0.040	0.139		0.197	−0.063
η*_1Hz_	0.013	0.463 **	0.384 **	0.215	−0.177	−0.437 **	−0.545 **	−0.679 **	0.997 **	0.430 **	0.997 **	0.984 **	−0.013		0.774 **
τ_y_	0.002	0.515 **	0.416 **	0.195	−0.167	−0.291 *	−0.224 *	−0.344 **	0.727 **	0.043	0.740 **	0.700 **	−0.188	0.732 **	

^1^ TP: total protein; Cas: caseins; TS: total solids; SCC: somatic cell count; RCT: rennet coagulation time; T_G’=20Pa_: time when G′ was equal to 20 Pa; G’_max_: storage modulus at the end of the coagulation kinetics measurement; I_Ε_: elasticity increment; G’_1Hz_: storage modulus at 1Hz; G’’_1Hz_: loss modulus at 1Hz; tanδ_1Hz_: loss tangent at 1Hz; η*_1Hz_: complex viscosity; τ_y_: apparent yield stress. * Correlation is significant at the 0.05 level (2-tailed). ** Correlation is significant at the 0.01 level (2-tailed).

**Table 2:** Stepwise linear regression analysis applied to the independent variables, milk composition and quality characteristics, and the dependent variables, rennet-induced milk coagulation parameters.

	Extensive System							Intensive System						
Dependent Variable	Model	Predictors	R	R^2^	R^2^ Change	F Change	Sig. F Change	Model	Predictors	R	R^2^	R^2^ Change	F Change	Sig. F Change
RCT ^1^	1	pH	0.480	0.231	0.231	21.312	<0.001	1	pH	0.502	0.252	0.252	27.669	<0.001
	2	pH, TP	0.613	0.376	0.145	16.292	<0.001	2	pH, Fat	0.574	0.329	0.077	9.247	0.003
T_G′=20Pa_	1	pH	0.448	0.201	0.201	17.878	<0.001	1	pH	0.501	0.251	0.251	27.552	<0.001
								2	pH, TS	0.592	0.350	0.098	12.261	<0.001
G′_max_	1	pH	0.456	0.208	0.208	18.687	<0.001	1	Cas	0.648	0.420	0.420	59.442	<0.001
	2	pH, TP	0.573	0.328	0.120	12.450	<0.001	2	Cas, pH	0.710	0.504	0.083	13.607	<0.001
								3	Cas, pH, TS	0.734	0.539	0.035	6.125	0.015
IE	1	pH	0.335	0.112	0.112	9.000	0.004	1	pH	0.324	0.105	0.105	9.626	0.003
G′_1Hz_	1	pH	0.452	0.204	0.204	18.217	<0.001	1	Cas	0.661	0.437	0.437	63.649	<0.001
	2	pH, TP	0.585	0.342	0.138	14.721	<0.001	2	Cas, pH	0.718	0.515	0.078	13.058	0.001
								3	Cas, pH, TS	0.739	0.546	0.031	5.482	0.022
G″_1Hz_	1	TP	0.525	0.275	0.275	26.976	<0.001	1	Cas	0.676	0.457	0.457	68.885	<0.001
	2	TP, pH	0.610	0.373	0.097	10.847	0.002	2	Cas, pH	0.725	0.526	0.069	11.850	0.001
								3	Cas, pH, TS	0.734	0.552	0.026	4.697	0.033
tanδ_1Hz_	1	TP	0.457	0.209	0.209	18.764	<0.001	1	Cas	0.324	0.105	0.105	9.649	0.003
	2	TP, pH	0.570	0.325	0.116	11.997	<0.001	2	Cas, SCC	0.416	0.173	0.068	6.625	0.012
η*_1Hz_	1	TP	0.463	0.214	0.214	19.362	<0.001	1	Cas	0.662	0.439	0.439	64.055	<0.001
	2	TP, pH	0.584	0.340	0.126	13.396	<0.001	2	Cas, pH	0.718	0.516	0.078	12.973	0.001
								3	Cas, pH, TS	0.739	0.547	0.031	5.397	0.023
τ_y_	1	TP	0.515	0.265	0.265	25.576	<0.001	1	Cas	0.689	0.475	0.475	74.221	<0.001
								2	Cas, pH	0.730	0.533	0.058	10.075	0.002

^1^ TP: total protein; Cas: caseins; TS: total solids; SCC: somatic cell count; RCT: rennet coagulation time; T_G’=20Pa_: time when G′ was equal to 20 Pa; G’_max_: storage modulus at the end of the coagulation kinetics measurement; I_Ε_: elasticity increment; G’_1Hz_: storage modulus at 1Hz; G’’_1Hz_: loss modulus at 1Hz; tanδ_1Hz_: loss tangent at 1Hz, η*_1Hz_: complex viscosity; τ_y_: apparent yield stress.

**Table 3:** Factor loadings for the principal component analysis (PCA) ^1^ applied to compositional, quality, and coagulation variables of milks from goats of the Skopelos breed raised under intensive and extensive farming systems.

	PC1 (30.6% ^2^)	PC2 (26.2%)	PC3 (12.6%)	PC4 (9.4%)
Fat		0.590	−0.753	
Cas ^3^		0.851	0.401	
TS		0.861	−0.449	
SCC				0.607
pH	0.665			
RCT	0.822			
T_G′=20Pa_	0.892			
G’_max_	−0.787	0.504		
I_E_	−0.550			0.496
tanδ_1Hz_		0.662		0.440
τ_y_	−0.587	0.441		

^1^ The Kaiser criterion (eigenvalue >1) was used to select the principal components. Factor loadings with communality values less than 0.4 were excluded. ^2^ Explained variance. ^3^ Cas: caseins; TS: total solids; SCC: somatic cell count; RCT: rennet coagulation time; T_G’=20Pa_: time when G′ was equal to 20 Pa; G’_max_: storage modulus at the end of the coagulation kinetic measurements; I_Ε_: elasticity increment; tanδ_1Hz_: loss tangent at 1 Hz, τ_y_: apparent yield stress.

## Data Availability

The original contributions presented in this study are included in the article/Supplementary Materials. Further inquiries can be directed to the corresponding author.

## Supplementary Materials

The following supporting information can be downloaded at: https://www.mdpi.com/article/10.3390/foods14081316/s1, Figure S1: Storage modulus, G′, of Skopelos goat milks collected from intensive and extensive farming systems at different sampling years (2022, 2023) as a function of renneting time; Table S1: *t*-Test analysis of coagulation rheological parameters of Skopelos goat milks collected from extensive farming systems during two different sampling years (2022 vs. 2023); Table S2: *t*-Test analysis of coagulation rheological parameters of Skopelos goat milks collected from intensive farming systems during two different sampling years (2022 vs. 2023); Table S3: *t*-Test analysis of coagulation rheological parameters of Skopelos goat milks collected from two different farming systems (intensive vs. extensive) during the 2022 and 2023 sampling years.

## Abbreviations

The following abbreviations are used in this manuscript:

RCT	Rennet coagulation time
SCC	Somatic cell count
PCA	Principal component analysis
