# Supplementary material for: Effects of Farming System on the Rheological Behavior of Rennet-Induced Coagulation in Milk from Skopelos Breed Goats
Source: Foods. 2025 Apr 10;14(8):1316. doi: 10.3390/foods14081316 (PMC12027251; doi:10.3390/foods14081316)
Supplement: Supplementary file 1 [file foods-14-01316-s001.zip › foods-3562720-supplementary.pdf]

Supplementary

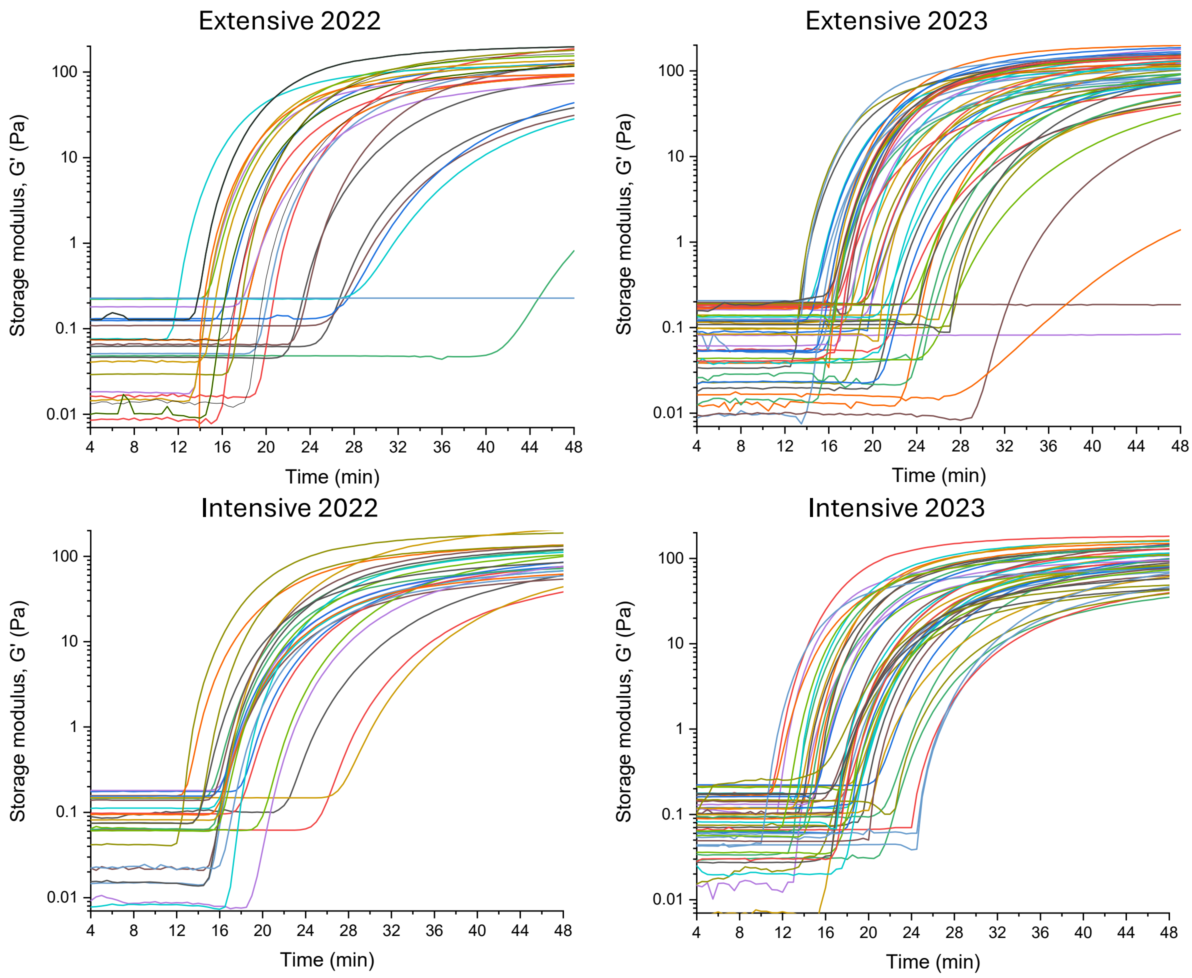

Figure S1: Storage modulus, G', of Skopelos goat milks collected from intensive and extensive farming systems at different sampling years (2022, 2023) as a function of renneting time.

Table S1. t-Test analysis of coagulation rheological parameters of Skopelos goat milks collected from extensive farming systems during two different sampling years (2022 vs. 2023).

Group Statistics

	Batch	N	Mean	Std. Deviation	Std. Error Mean
RCT (min)	Exte 2022	27	17.6296	3.98698	.76729
	Exte 2023	46	18.8804	4.06227	.59895
T (min) at G'=20Pa	Exte 2022	27	24.8704	6.19179	1.19161
	Exte 2023	46	26.8152	6.82578	1.00641
G'max (Pa)	Exte 2022	27	127.3815	44.70022	8.60256
	Exte 2023	46	108.2217	43.27440	6.38046
Ie (min-1)	Exte 2022	27	.7470	.23033	.04433
	Exte 2023	46	.6618	.22363	.03297
G' (Pa) at 1 Hz	Exte 2022	27	132.3259	45.48694	8.75397
	Exte 2023	46	112.7196	43.60378	6.42903
G" (Pa) at 1 Hz	Exte 2022	27	42.3963	14.72537	2.83390
	Exte 2023	46	38.6243	15.46930	2.28082
tand at 1 Hz	Exte 2022	27	.3212	.01912	.00368
	Exte 2023	46	.3415	.02198	.00324
Compl. Viscosity (Pa.s) at 1 Hz	Exte 2022	27	20.9278	7.19811	1.38528
	Exte 2023	46	18.0554	7.01671	1.03456
Yield Stress (Pa)	Exte 2022	27	6.5652	2.15541	.41481
	Exte 2023	46	5.7609	2.16163	.31872

		Levene's Test for Equality of Variances		t-test for Equality of Means							
						Significance				95% Confidence Interval of the Difference	
		F	Sig.	t	df	One-Sided p	Two-Sided p	Mean Difference	Std. Error Difference	Lower	Upper
RCT (min)	Equal variances assumed	.320	.573	-1.279	71	.103	.205	-1.25081	.97820	-3.20129	.69968
	Equal variances not assumed			-1.285	55.445	.102	.204	-1.25081	.97339	-3.20116	.69955
T (min) at G'=20Pa	Equal variances assumed	1.100	.298	-1.215	71	.114	.228	-1.94485	1.60026	-5.13567	1.24597
	Equal variances not assumed			-1.247	58.982	.109	.217	-1.94485	1.55974	-5.06590	1.17621
G'max (Pa)	Equal variances assumed	.141	.708	1.804	71	.038	.075	19.15974	10.61925	-2.01443	40.33392
	Equal variances not assumed			1.789	53.176	.040	.079	19.15974	10.71048	-2.32110	40.64058
Ie (min-1)	Equal variances assumed	.005	.943	1.555	71	.062	.124	.08526	.05482	-.02404	.19457
	Equal variances not assumed			1.543	53.303	.064	.129	.08526	.05525	-.02553	.19606
G' (Pa) at 1 Hz	Equal variances assumed	.055	.816	1.825	71	.036	.072	19.60636	10.74065	-1.80989	41.02261
	Equal variances not assumed			1.805	52.745	.038	.077	19.60636	10.86114	-2.18078	41.39350
G" (Pa) at 1 Hz	Equal variances assumed	.872	.354	1.024	71	.155	.310	3.77195	3.68532	-3.57638	11.12028
	Equal variances not assumed			1.037	56.818	.152	.304	3.77195	3.63774	-3.51300	11.05690
tand at 1 Hz	Equal variances assumed	.645	.425	-3.991	71	<.001	<.001	-.02030	.00509	-.03044	-.01016
	Equal variances not assumed			-4.140	60.846	<.001	<.001	-.02030	.00490	-.03011	-.01049
Compl. Viscosity (Pa.s) at 1 Hz	Equal variances assumed	.174	.678	1.673	71	.049	.099	2.87234	1.71735	-.55196	6.29665
	Equal variances not assumed			1.661	53.479	.051	.103	2.87234	1.72896	-.59479	6.33947
Yield Stress (Pa)	Equal variances assumed	.156	.695	1.536	71	.064	.129	.80432	.52351	-.23953	1.84816
	Equal variances not assumed			1.538	54.737	.065	.130	.80432	.52311	-.24414	1.85277

Table S2. t-Test analysis of coagulation rheological parameters of Skopelos goat milks collected from intensive farming systems during two different sampling years (2022 vs. 2023).

Group Statistics

	Batch	N	Mean	Std. Deviation	Std. Error Mean
RCT (min)	Inte 2022	39	16.5641	2.71249	.43435
	Inte 2023	45	16.6889	3.54402	.52831
T (min) at G'=20Pa	Inte 2022	39	25.2872	4.67446	.74851
	Inte 2023	45	25.5867	6.15392	.91737
G'max (Pa)	Inte 2022	39	99.9179	36.98305	5.92203
	Inte 2023	45	96.8022	37.92969	5.65422
Ie (min-1)	Inte 2022	39	.7151	.27823	.04455
	Inte 2023	45	.6460	.20460	.03050
G' (Pa) at 1 Hz	Inte 2022	39	103.9821	37.82249	6.05644
	Inte 2023	45	100.3444	38.32568	5.71326
G'' (Pa) at 1 Hz	Inte 2022	39	30.9872	11.87027	1.90076
	Inte 2023	45	30.3444	11.95379	1.78197
tand at 1 Hz	Inte 2022	39	.2966	.01530	.00245
	Inte 2023	45	.3017	.01473	.00220
Compl. Viscosity (Pa.s) at 1 Hz	Inte 2022	39	16.3562	5.97138	.95619
	Inte 2023	45	15.7964	6.04911	.90175
Yield Stress (Pa)	Inte 2022	39	6.1615	2.08536	.33393
	Inte 2023	45	5.1578	1.81915	.27118

		Levene's Test for Equality of Variances		t-test for Equality of Means							
						Significance				95% Confidence Interval of the Difference	
		F	Sig.	t	df	One-Sided p	Two-Sided p	Mean Difference	Std. Error Difference	Lower	Upper
RCT (min)	Equal variances assumed	3.586	.062	-.179	82	.429	.858	-.12479	.69697	-1.51129	1.26172
	Equal variances not assumed			-.182	80.826	.428	.856	-.12479	.68394	-1.48565	1.23608
T (min) at G'=20Pa	Equal variances assumed	3.772	.056	-.248	82	.402	.805	-.29949	1.20718	-2.70095	2.10198
	Equal variances not assumed			-.253	80.682	.400	.801	-.29949	1.18399	-2.65541	2.05643
G'max (Pa)	Equal variances assumed	.290	.592	.380	82	.353	.705	3.11573	8.20281	-13.2023	19.43373
	Equal variances not assumed			.381	80.841	.352	.705	3.11573	8.18784	-13.1760	19.40744
Ie (min-1)	Equal variances assumed	3.813	.054	1.308	82	.097	.195	.06909	.05284	-.03602	.17421
	Equal variances not assumed			1.280	68.896	.102	.205	.06909	.05399	-.03862	.17681
G' (Pa) at 1 Hz	Equal variances assumed	.278	.599	.436	82	.332	.664	3.63761	8.33393	-12.9412	20.21645
	Equal variances not assumed			.437	80.600	.332	.663	3.63761	8.32597	-12.9297	20.20493
G'' (Pa) at 1 Hz	Equal variances assumed	.134	.715	.247	82	.403	.806	.64274	2.60676	-4.54294	5.82841
	Equal variances not assumed			.247	80.468	.403	.806	.64274	2.60544	-4.54179	5.82726
tand at 1 Hz	Equal variances assumed	.819	.368	-1.532	82	.065	.129	-.00503	.00328	-.01155	.00150
	Equal variances not assumed			-1.528	79.344	.065	.131	-.00503	.00329	-.01157	.00152
Compl. Viscosity (Pa.s) at 1 Hz	Equal variances assumed	.257	.613	.425	82	.336	.672	.55971	1.31555	-2.05734	3.17676
	Equal variances not assumed			.426	80.594	.336	.671	.55971	1.31432	-2.05558	3.17500
Yield Stress (Pa)	Equal variances assumed	.062	.804	2.356	82	.010	.021	1.00376	.42597	.15637	1.85115
	Equal variances not assumed			2.333	76.075	.011	.022	1.00376	.43017	.14702	1.86051

Table S3. t-Test analysis of coagulation rheological parameters of Skopelos goat milks collected from two different farming systems (intensive vs. extensive) during the 2022 and 2023 sampling years.

Group Statistics

	Farming System	N	Mean	Std. Deviation	Std. Error Mean
RCT (min)	Exte 2022-23	73	18.4178	4.05262	.47432
	Inte 2022-23	84	16.6310	3.16715	.34556
T (min) at G'=20Pa	Exte 2022-23	73	26.0959	6.62251	.77511
	Inte 2022-23	84	25.4476	5.48658	.59863
G'max (Pa)	Exte 2022-23	73	115.3082	44.48264	5.20630
	Inte 2022-23	84	98.2488	37.30020	4.06979
Ie (min-1)	Exte 2022-23	73	.6933	.22833	.02672
	Inte 2022-23	84	.6781	.24256	.02647
G' (Pa) at 1 Hz	Exte 2022-23	73	119.9712	45.01449	5.26855
	Inte 2022-23	84	102.0333	37.90711	4.13600
G" (Pa) at 1 Hz	Exte 2022-23	73	40.0195	15.20612	1.77974
	Inte 2022-23	84	30.6429	11.84755	1.29267
tand at 1 Hz	Exte 2022-23	73	.3340	.02305	.00270
	Inte 2022-23	84	.2993	.01512	.00165
Compl. Viscosity (Pa.s) at 1 Hz	Exte 2022-23	73	19.1178	7.17155	.83937
	Inte 2022-23	84	16.0563	5.98347	.65285
Yield Stress (Pa)	Exte 2022-23	73	6.0584	2.17966	.25511
	Inte 2022-23	84	5.6238	1.99974	.21819

		Levene's Test for Equality of Variances		t-test for Equality of Means							
						Significance		Mean Difference	Std. Error Difference	95% Confidence Interval of the Difference	
		F	Sig.	t	df	One-Sided p	Two-Sided p			Lower	Upper
RCT (min)	Equal variances assumed	4.635	.033	3.097	155	.001	.002	1.78686	.57694	.64718	2.92653
	Equal variances not assumed			3.045	135.581	.001	.003	1.78686	.58685	.62629	2.94743
T (min) at G'=20Pa	Equal variances assumed	1.562	.213	.671	155	.252	.503	.64827	.96660	-1.26114	2.55768
	Equal variances not assumed			.662	140.230	.255	.509	.64827	.97936	-1.28796	2.58450
G'max (Pa)	Equal variances assumed	2.103	.149	2.613	155	.005	.010	17.05941	6.52749	4.16508	29.95374
	Equal variances not assumed			2.582	141.156	.005	.011	17.05941	6.60823	3.99552	30.12330
Ie (min-1)	Equal variances assumed	.029	.865	.402	155	.344	.688	.01520	.03777	-.05942	.08981
	Equal variances not assumed			.404	153.992	.343	.687	.01520	.03761	-.05910	.08949
G' (Pa) at 1 Hz	Equal variances assumed	1.922	.168	2.710	155	.004	.007	17.93790	6.61816	4.86447	31.01133
	Equal variances not assumed			2.678	141.478	.004	.008	17.93790	6.69807	4.69666	31.17913
G" (Pa) at 1 Hz	Equal variances assumed	6.772	.010	4.337	155	<.001	<.001	9.37659	2.16205	5.10572	13.64747
	Equal variances not assumed			4.263	135.333	<.001	<.001	9.37659	2.19966	5.02645	13.72674
tand at 1 Hz	Equal variances assumed	15.513	<.001	11.280	155	<.001	<.001	.03468	.00307	.02861	.04075
	Equal variances not assumed			10.967	121.181	<.001	<.001	.03468	.00316	.02842	.04094
Compl. Viscosity (Pa.s) at 1 Hz	Equal variances assumed	2.526	.114	2.916	155	.002	.004	3.06150	1.05002	.98731	5.13569
	Equal variances not assumed			2.879	140.773	.002	.005	3.06150	1.06337	.95927	5.16373
Yield Stress (Pa)	Equal variances assumed	.454	.501	1.302	155	.097	.195	.43455	.33366	-.22456	1.09366
	Equal variances not assumed			1.294	147.429	.099	.198	.43455	.33569	-.22884	1.09793
